# Association between soluble angiotensin-converting enzyme 2 in saliva and SARS-CoV-2 infection: a cross-sectional study

**DOI:** 10.1038/s41598-023-31911-2

**Published:** 2023-04-12

**Authors:** Samuel Bru, Pedro Brotons, Iolanda Jordan, Laia Alsina, Desiree Henares, Reyes Carballar, Mariona Fernandez de Sevilla, Irene Barrabeig, Victoria Fumado, Bàrbara Baro, Joan Marc Martínez-Láinez, Juan J. Garcia-Garcia, Quique Bassat, Albert Balaguer, Josep Clotet, Cristian Launes, Carmen Muñoz-Almagro

**Affiliations:** 1 Department of Basic Sciences, School of Medicine and Health Sciences, Universitat Internacional de Catalunya, Inmaculada, 22, 28029 Barcelona, Spain; 2 Institut de Recerca Sant Joan de Déu, Santa Rosa, 39-57, Esplugues de Llobregat, 08950 Barcelona, Spain; 3 Department of Medicine, School of Medicine and Health Sciences, Universitat Internacional de Catalunya, Inmaculada, 22, 28029 Barcelona, Spain; 4 Consorcio de Investigación Biomédica en Red de Epidemiología y Salud (CIBERESP), Instituto de Salud Carlos III, Monforte de Lemos 3-5, 28029 Madrid, Spain; 5 Paediatric Intensive Care Unit, Hospital Sant Joan de Déu, Sant Joan de Déu 2, 08950 Esplugues de Llobregat, Spain; 6 Paediatric Allergy and Clinical Immunology Department, Hospital Sant Joan de Déu, Sant Joan de Déu 2, 08950 Esplugues de Llobregat, Spain; 7 Paediatrics Department, Hospital Sant Joan de Déu, Sant Joan de Déu 2, 08950 Esplugues de Llobregat, Spain; 8 Agència de Salut Pública de Catalunya, Roc Boronat 81, 08005 Barcelona, Spain; 9 Infectious Diseases Department, Hospital Sant Joan de Déu, Sant Joan de Déu 2, 08950 Esplugues de Llobregat, Spain; 10 ISGlobal, Hospital Clínic-Universitat de Barcelona, Rosselló 132, 08036 Barcelona, Spain; 11 Centro de Investigação em Saúde de Manhiça (CISM), Manhiça, Rua 12, 1229 Manhiça, Mozambique; 12 Institució Catalana de Recerca i Estudis Avançats (ICREA), Lluís Companys 23, 08010 Barcelona, Spain

**Keywords:** Viral infection, Molecular medicine

## Abstract

This study aimed to investigate the association between saliva soluble angiotensin-converting enzyme 2 (sACE2) and severe acute respiratory syndrome coronavirus 2 (SARS-CoV-2) infection in children and adults**.** We selected a convenience sample of adults with post-acute SARS-CoV-2 infection and their household children living in quarantined family households of the metropolitan Barcelona region (Spain) during the spring 2020 pandemic national lockdown. Participants were tested for saliva sACE2 quantification by western blot and nasopharyngeal SARS-CoV-2 RT-PCR detection. A total of 161 saliva samples [82 (50.9%) from children; 79 (49.1%) from females] yielded valid western blot and RT-PCR results. Saliva sACE2 was detected in 79 (96.3%) children and 76 (96.2%) convalescent adults. Twenty (24.4%) children and 20 (25.3%) convalescent adults were positive for SARS-CoV-2 in nasopharynx by RT-PCR. SARS-CoV-2 RT-PCR-negative children had a significantly higher mean proportional level of saliva sACE2 (0.540 × 10^–3^%) than RT-PCR-positive children (0.192 × 10^–3^%, *p* < 0.001) and convalescent adults (0.173 × 10^–3^%, *p* < 0.001). In conclusion, children negative for nasopharyngeal SARS-CoV-2 RT-PCR appear to exhibit a higher concentration of saliva sACE2 than SARS-CoV-2 RT-PCR-positive children and convalescent adults. Release of adequate levels of sACE2 in saliva could play a protective role against SARS-CoV-2.

## Introduction

The coronavirus disease 2019 (COVID-19) pandemic caused by the severe acute respiratory syndrome coronavirus 2 (SARS-CoV-2) remains a significant global health threat, accounting for over 581 million confirmed cases and more than 6.4 million deaths, as of 8 August 2022^1^. The angiotensin-converting enzyme 2 (ACE2), a protein that plays a pivotal role as a key regulator of the renin–angiotensin–aldosterone system^2^, has been identified as the cell entry receptor of SARS-CoV-2^3^. ACE2 is ubiquitously expressed in different tissues and particularly abundant in the lung, the intestine, and the kidney^4,5^. Recent in vitro studies have suggested that it may also be highly enriched in epithelial cells of the oral cavity, an accessible portal for SARS-CoV-2 entry and cell binding^6,7^. A soluble form of ACE2 (sACE2) is released either constitutively or induced by inflammation in small amounts in the blood and other body fluids^8^. While tissue-specific ACE2 is characterized by full-length isoforms, the presence of both full- and short-length isoforms of sACE2 has been detected in plasma^9^ and urine^10,11^.

Saliva, a fluid largely generated from salivary glands in the oral cavity, enables simple, safe, and convenient sample collection for detection and measurement of sACE2 and other biomarkers. Little is known about the role of saliva sACE2 in SARS-CoV-2 infection. The objective of this study was to explore the association between sACE2 release in saliva and nasopharyngeal (NP) SARS-CoV-2 detection by reverse transcriptase polymerase chain reaction (RT-PCR) in children and adults**.**

## Methods

### Study design

This cross-sectional study was nested within a prospective population-based study that was conducted with quarantined family households in the metropolitan region of Barcelona (Spain) during the spring 2020 pandemic national lockdown^12^. The main study included adults that were recovering from acute COVID-19 in their homes as well as their children aged less than 15 years cohabiting in the same household. Adults’ SARS-CoV-2 infection had been confirmed by RT-PCR in nasopharynx at least 15 days before recruitment. Research nurses of University Hospital Sant Joan de Déu (HSJD) in Barcelona visited the households and collected relevant data of family members including age, sex, body mass index (BMI), smoking habit, and adult previous in- or out-of-hospital care (as a proxy for COVID-19 severity). Saliva and NP specimens were taken from individuals for further analysis.

A convenience sample of children and adults was selected among those that participated in the main study according to a 1:1 ratio between children and adults, males and females, and SARS-CoV-2 RT-PCR-positive and negative individuals. The study outcomes were saliva sACE2 detection and quantification in saliva by western blot and NP detection of SARS-CoV-2 and other respiratory viruses by RT-PCR.

### Saliva and nasopharyngeal sample collection and storage

Saliva was collected either in the first hour of the morning before breakfast and teeth brushing to maximize the detection yield of SARS-CoV-2 RT-PCR^13^ or after a minimum time lag of one hour since last food intake or last teeth brushing to homogenize the conditions of sampling. Micropipettes were used to collect infants’ saliva from their mouth and transfer it to sterile tubes. Older children and adults directly spitted their saliva into the tubes. NP swabs were collected as previously described^14^.

Saliva and NP swabs were transported in ambient temperature and were received and stored at −80 °C in the biobank of HSJD. Specimens with insufficient volume or that yielded invalid results for sACE2 or SARS-CoV-2 RNA detection were excluded from analysis.

### Quantification of saliva sACE2 concentration and isoform identification

Saliva specimens were tested for detection and quantification of sACE2 isoforms in the School of Medicine and Health Sciences of Universitat Internacional de Catalunya (UIC) in Barcelona. In essence, a series of protein separation, incubation and washing steps were performed after previous virus inactivation by heating. Protein concentration was quantified using different amounts of ACE2 as standard. Measurement of the total protein concentration was calculated using the Bradford protein assay^15^. The detailed procedure of saliva sACE2 quantification and identification of isoforms is specified in the Supplemental File.

### Detection of SARS-CoV-2 and other respiratory viruses in nasopharynx

Procedures for detection of SARS-CoV-2 RNA and RNA/DNA detection of other respiratory viruses in NP swabs were carried out in the clinical laboratory of HSJD^14^. In brief, SARS-CoV-2 RT-PCR was performed according to the CDC-006-00019 protocol, which targets detection of N1, N2 SARS-Cov2 genes and human RNAse P gene as internal control. Allplex Respiratory Panels 1, 2, and 3 (Seegene Inc., Seoul, Korea) were used for RT-PCR detection of adenovirus (AdV), bocavirus (BoV) types 1/2/3/4, coronavirus (CoV) types 229E/NL63/OC43, influenza A virus (IFV-A) including differentiation of subtypes H1/H1N1-2009/H3, influenza B virus (IFV-B), metapneumovirus (MPV), parainfluenza virus (PIV) types 1/2/3/4, respiratory syncytial virus (RSV) types A/B, and rhinovirus/enterovirus (RV/EV).

### Statistical analysis

Participants were grouped by age into children and adults. A BMI cut-off value of 30 kg/m^2^ was established to distinguish between normal weight and overweight. Soluble ACE level in each saliva sample was normalized to that of total protein level in the sample and expressed as a proportion. ACE2 isoforms were categorized into short-length and full-length isoforms according to a molecular weight cut-off of 90 kDa. The association between detection of saliva sACE2 and SARS-CoV-2 was assessed using the chi-square or the Fisher’s exact test. Proportional saliva sACE2 level was evaluated in SARS-CoV-2 RT-PCR-positive and negative children and convalescent adults using the t-Student test or the Mann–Whitney test. Statistical significance was set at a *p*-value of < 0.05 and variability of outcomes was reported with 95% confidence intervals (CIs). Statistical analyses were performed using Stata v.15 software (StataCorp, College Station, TX, US).

### Ethics statement

Adults and children’s parents or legal representatives gave their informed consent for sample donation to the HSJD bio bank, which is accredited as a research bio repository integrated into the Spanish Biobank Network of the national Instituto de Salud Carlos III. The Ethics Committee of HSJD approved the main intra-household surveillance study, including the use of bio-banked samples for further studies nested within it like the present one. All participant identification data were duly de-identified. All experiments were performed in accordance with the relevant guidelines and regulations.

## Results

### Characteristics of the study population

A total of 161 participants with valid SARS-CoV-2 RT-PCR and sACE2 results for saliva were selected for the study. Of them, 82 (50.9%) were collected from children and 79 (49.1%) from adults. A proportion of 44/82 (57.1%) of children and 33/79 (42.9%) of adults were male. Thirty (38.0%) adults were recovering at home after previous hospitalisation due to severe COVID-19. The implementation of the convenience sampling inclusion criteria resulted in the selection of 66/161 (41,0%) of participants that were members of the same household familiar unit, including one adult and one child in 20 households, two children in eight households, one adult and two children in three households, and one adult and three children in one household. Table 1 depicts epidemiological characteristics of the study population.

**Table 1:** Epidemiological characteristics of study population.

Characteristic	Children (n = 82)	Adults (n = 79)
Median age (IQR), yr	4.7 (3.5–8.6)	39.0 (36.6–43.5)
Sex, male	44 (57.1)	33 (42.9)
Median BMI (IQR), kg/m^2^ (total n = 152)	16.7 (15.4–18.1)	24.1 (21.9–27.6)
Weight status, overweight (total n = 152)	–	17 (21.5)
Smoking habit (total n = 154)	–	8 (10.1)

Values expressed as counts (%) unless otherwise stated.

*IQR* interquartile range, *yr* years, *BMI* body mass index.

### Saliva sACE2 detection and quantification

**Figure 1:**
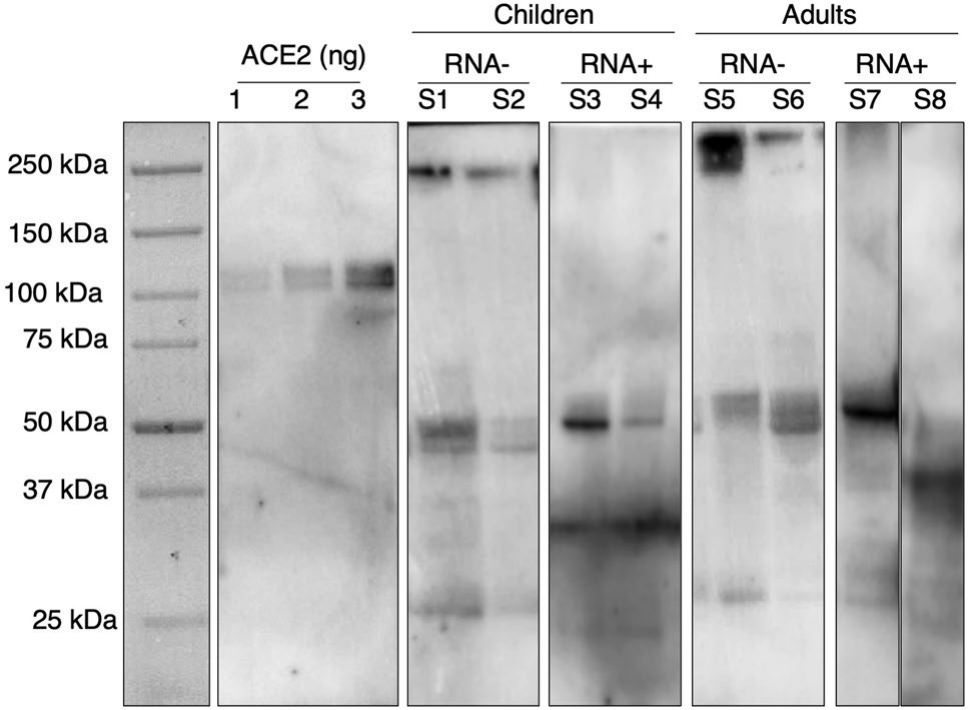
Western blot image showing sACE2 species in children and adults. First panel: molecular weight marker. Second panel: different amount of sACE2 used as standard. Rest of panels: representative distribution of sACE2 forms according to age group and infection status. *RNA*− non infected, RNA+ infected.

Soluble ACE2 was detected in 155 (96.3%) out of 161 saliva samples, including 79 (96.3%) specimens sampled from children and 76 (96.2%) from adults. Western blot analysis showed a complex picture, confirming the presence of several circulating sACE2 species in saliva that were characterized by different molecular weights: a high molecular weight ranging from 175 to < 250 kDa that might correspond to full-length highly-glycosylated forms^16^, full-length partially-glycosylated forms ranging from 90 to 150 kDa, depending on the number of post-translational modifications^17^, and different bands ranging from 75 up to 90 kDa that have been identified as de-glycosylated forms from full-length sACE2^10^. The most prevalent immune-reactive bands in saliva spanned from 50 to 70 kDa and might correspond to the described splicing variant isoform of ACE2^18^. We also detected other multiple bands of low molecular weight ranging from 16 to 40 kDa which could be the result of the high proteolytic activity present in the saliva environment^19^. Details of saliva sACE2 isoform distribution in children and adults according to molecular weight ranges are provided in Table 2. Figure 1 includes a representative image of western blot sACE2 bands in saliva according to age group and infection status.

**Table 2:** Saliva sACE2 isoform identification in children and adults.

sACE2 isoform type	SARS-CoV-2 RNA− children		SARS-CoV-2 RNA+ children		Convalescent adults			
	No. (%)	Mean sACE2 level (A)	No. (%)	Mean sACE2 level (B)	No. (%)	Mean sACE2 level (C)	p-value (A–B)	p-value (A–C)
Short-length (< 90 kDa)	179 (86.5)	0.393	60 (96.8)	0.189	253 (93.4)	0.168	0.05	< 0.001
De-glycosylated (75–87 kDa)	7 (3.4)		2 (3.2)		4 (1.5)			
Splicing variant (50–70 kDa)	106 (51.2)		24 (38.7)		90 (33.2)			
Proteolytic product (< 50 kDa)	66 (31.9)		34 (54.8)		159 (58.7)			
Full-length (≥ 90 kDa)	28 (13.5)	0.449	2 (3.2)	0.027	18 (6.6)	0.157	0.32	0.09
Highly glycosylated (≥ 175 kDa)	20 (9.7)		2 (3.2)		8 (3.0)			
Partially glycosylated (90–150 kDa)	8 (3.9)		–		10 (3.7)		–	

Mean sACE2 level values expressed as proportions (10^–3^%) of total proteins contained in saliva samples.

*SARS-CoV-2* acute respiratory syndrome coronavirus 2, *sACE2* soluble angiotensin-converting enzyme 2.

### Detection of SARS-CoV-2 and other respiratory viruses

Twenty (24.4%) out of 82 children had a positive NP SARS-CoV2 RT-PCR result. All of them were asymptomatic. Among the group of 79 convalescent adults, 59 (74.7%) cleared the virus whereas 20 (25.3%) remained persistently positive by RT-PCR. Mean NP SARS-CoV-2 RNA load was 3.6 log_10_ copies/mL (SD, 0.9) in children, 3.7 log_10_ copies/mL (SD, 1.3) in adults previously hospitalized, and 3.3 log_10_ copies/mL (SD, 0.9) in adults not previously hospitalized. No statistically differences were found in mean NP SARS-CoV-2 RNA load between children and adults (*p* = 0.99) or between adults previously hospitalized and not hospitalized (*p* = 0.44). Thirty-seven (23.3%) out of 159 NP samples with valid results for the RT-PCR respiratory panel were positive, including 29 specimens from children and 8 from adults. A total of 43 respiratory viruses other than SARS-CoV-2 were identified, including rhinovirus-enterovirus (RV/EV) (n = 29), bocavirus (n = 9), other coronaviruses (n = 2), parainfluenza virus 1 (n = 2), and adenovirus (n = 1). Eighteen NP samples positive for SARS-CoV-2 were co-infected with one (n = 15) or two respiratory viruses (n = 3). RV/EV was the main species involved in co-infection with SARS-CoV-2 (n = 15). The distribution of SARS-CoV-2 and other respiratory viruses in children and adults is shown in Table 3.

**Table 3:** Distribution of SARS-CoV-2 and other respiratory viruses in children and adults.

Characteristic	Children (n = 82)	Adults (n = 79)	p-value
SARS-CoV-2 RT-PCR result			
Negative	62 (75.6)	–	–
Positive	20 (24.4)	–	–
Negativized after ≥ 15 days	–	59 (74.7)	–
Persistently positive after ≥ 15 days	–	20 (25.3)	–
RT-PCR respiratory viral panel (children, n = 81; adults, n = 78)	29 (35.8)	8 (10.3)	< 0.001
Rhinovirus–enterovirus	22 (27.2)	7 (9.0)	< 0.01
Bocavirus	7 (8.6)	2 (2.6)	0.10
Coronavirus 229E/NL63/OC43	1 (1.2)	1 (1.3)	0.98
Parainfluenza virus 1	2 (2.5)	–	–
Adenovirus	1 (1.2)	–	–

*SARS-CoV-2* acute respiratory syndrome coronavirus 2, *RT-PCR* reverse-transcriptase polymerase-chain reaction.

### Association of sACE2 with SARS-CoV-2 and other variables

Detection of saliva sACE2 was significantly associated with weight status (normal weight: 132/135, 97.8%; overweight: 15/17, 88.4%; *p* = 0.04) but not with age group (children: 79/82, 96.3%; adults: 76/79, 96.2%; *p* = 0.96), sex (males: 75/77, 97.4%; females: 80/84, 95.2%; *p* = 0.47), smoking habit (smokers: 8/8, 100.0%; non-smokers: 140/146, 95.9%; *p* = 0.56), SARS-CoV-2 RT-PCR detection (negative: 118/121, 97.5%; positive: 37/40, 92.5%; *p* = 0.15), or detection of respiratory viruses different from SARS-CoV-2 (negative: 118/122, 96.7%; positive: 35/37, 94.6%; *p* = 0.55).

**Figure 2:**
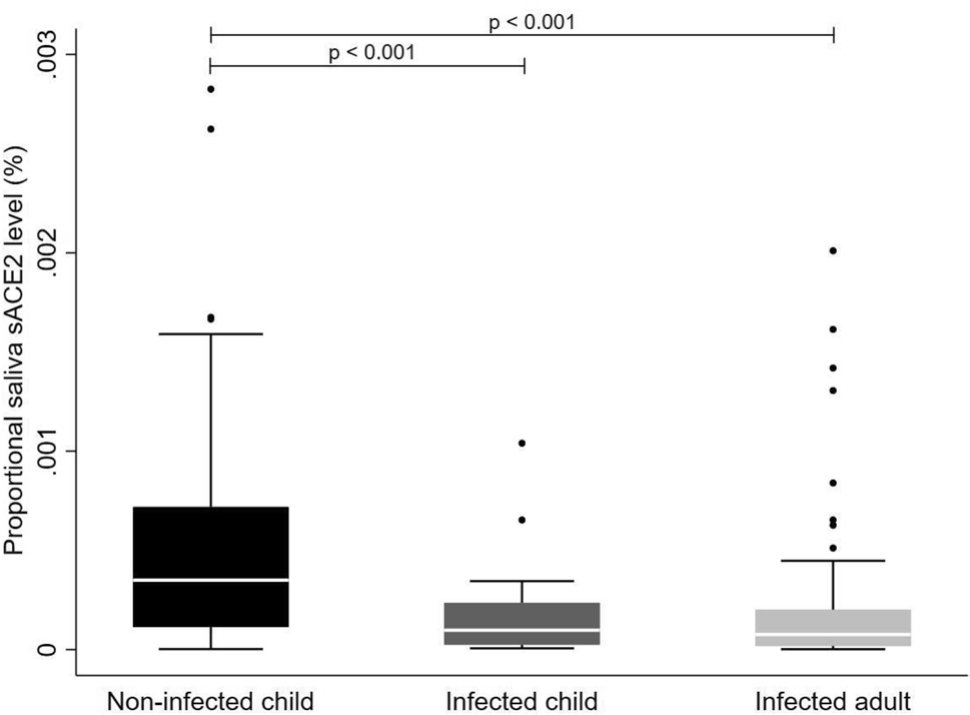
Proportional sACE2 concentration in saliva of children and adults.

Saliva of SARS-CoV-2 RT-PCR-negative children had a significantly higher mean proportional level of sACE2 (0.540 × 10^–3^%) than that of SARS-CoV-2 RT-PCR-positive children (0.192 × 10^–3^%, *p* < 0.001) and convalescent adults (0.173 × 10^–3^%, *p* < 0.001). Mean proportional level attributable to saliva short-length sACE2 isoforms (< 90 kDa) was markedly greater in children negative by SARS-CoV-2 RT-PCR (0.393 × 10^–3^%, *p* < 0.001) than in convalescent adults (0.168 × 10^–3^%, *p* < 0.001) and also tended to be significantly higher than in RT-PCR positive children (0.189 × 10^–3^%, *p* = 0.05). In turn, there were no statistically significant differences in mean proportional saliva sACE2 level corresponding to full-length isoforms ≥ 90 kDa between children negative for SARS-CoV-2 RT-PCR (0.449 × 10^–3^%), SARS-CoV-2 RT-PCR-positive children (0.027 × 10^–3^%, *p* = 0.32), and convalescent adults (0.157 × 10^–3^%, *p* = 0.09). Figure 2 represents proportional sACE2 levels in saliva of children with positive and negative SARS-CoV-2 detection and convalescent adults.

Proportional sACE2 level in saliva was not significantly associated with sex (mean value of 0.371 × 10^–3^% in females vs. 0.291 × 10^–3^% in males, *p* = 0.31), weight status (0.340 × 10^–3^% for normal weight vs. 0.160 × 10^–3^% for overweight, *p* = 0.18), smoking habit (0.189 × 10^–3^% in non-smokers vs. 0.303 × 10^–3^% in smokers, *p* = 0.42), or detection of respiratory viruses other than SARS-CoV-2 (0.352 × 10^–3^% in positives vs. 0.324 × 10^–3^% in negatives, *p* = 0.77). Differences in mean proportional sACE2 concentration in saliva between adults that experienced COVID-19 out of the hospital and those that required hospitalisation were not significant, either (0.230 × 10^–3^% vs. 0.149 × 10^–3^%, *p* = 0.37).

## Discussion

Our study provides initial evidence that levels of saliva sACE2 were significantly higher in children with a negative SARS-CoV-2 RT-PCR result than in SARS-CoV-2 RT-PCR-positive children and convalescent adults. This finding suggests that release of adequate concentrations of sACE2 in saliva could have a protective effect from SARS-CoV-2. Specifically, short-length sACE2 isoforms of molecular weight < 90 kDa were distinctively identified in saliva of those children that were free from the virus and mostly contributed to the greater concentration of sACE2 measured in this specific age group.

Our observation of high sACE2 concentration in saliva of SARS-CoV-2 RT-PCR-negative children could be suggestive of a mechanism of action by which sACE2 shed constitutively in body fluids acts as a competitive interceptor of SARS-CoV-2 by blocking virus binding to full-length tissue-bound ACE2. Indeed, human recombinant sACE2 was described to produce inhibitory effects on severe COVID-19^20,21^. It was also documented that transcription of NP sACE2 was negatively correlated with SARS-CoV-2 RNA load in infected adults^22^. Increased levels of sACE2 in saliva of children free of the virus could contribute to explain why COVID-19 severity rises substantially with age^23,24^. An alternative mechanism of action has been hypothesised that would explain the release of sACE2 as an inflammatory response to virus acquisition and disease development. In this regard, a recent cross-sectional study with individuals of all ages reported a positive correlation of sACE2 activity in saliva with susceptibility to SARS-CoV-2 infection in children and adults as well as with disease severity in adults^25^. Of note, that study was based on the use of an enzymatic colorimetric assay to measure sACE2 activity. We speculate that the differences between our results and those of that study could be attributable, at least in part, to the different methods of measurement of sACE2 that were respectively implemented.

The interactions of specific sACE2 isoforms with SARS-CoV-2 and their relevance in the pathogenesis of COVID-19 are not well known. We identified short-length isoforms < 90 kDa that were much more abundant in saliva of children negative by SARS-CoV-2 RT-PCR than in that of SARS-CoV-2 RT-PCR-positive children or convalescent adults. A previous study also reported the characteristic presence of short-length sACE2 isoforms of ~ 70 and ~ 75 kDa in saliva of non-infected adults using western blot, which points to the plausibility that these saliva short-length isoforms may not only contribute to protect children but also adults from SARS-CoV-2^26^.

Interestingly, no relationship was revealed between detection of saliva sACE2 and detection of respiratory viruses different from SARS-CoV-2 and, in particular, RV. This result contrasts with the report of a previous study that showed substantial upregulation of ACE2 in airway epithelia of healthy and asthmatic patients in response to rhinovirus infection^27^. ACE2 is known to be involved in cytokine surge, which has been associated with severe COVID-19^28^ whereas RV infections have been related with the production of pro-inflammatory chemokines, a specific group within the family of cytokines, and might play a role in the pathogenesis of exacerbations of lower-airway diseases^29^. Contrasting results about the potential association between RV and sACE2 detection might be explained by differences in the type of ACE2 targeted (soluble *vs*. tissue-specific) and the type of sample analysed (saliva *vs*. nasal tissue) in each study, among other possible factors.

The present study has several strengths and limitations. A main strength is the utilization of western blot for identifying and measuring full- and short-length sACE2 isoforms, which brings an advantage over previous studies based on enzyme immunosorbent assay (ELISA) for quantification of sACE2 levels without isoform discrimination. An additional strength is the detection of a wide range of respiratory viruses apart from SARS-CoV-2 to assess their association with sACE2. We also acknowledge a number of limitations. First, the study does not allow to confirm whether sACE2 release in saliva is produced constitutively or as an induced host response to SARS-CoV-2 due to its exploratory cross-sectional design. Second, a non-randomised convenience sample was used, which does not preclude potential selection bias. Third, saliva was collected from adults after the acute infection phase. Even so, we were able to find marked differences in saliva sACE2 concentration between children negative by SARS-CoV-2 RT-PCR and convalescent adults presumably sampled beyond the viral load peak after 14 days of symptoms. On the other hand, we did not evaluate adult-to-children transmission of the virus. However, since differences in mean NP SARS-CoV-2 RNA load between adults previously hospitalized and not hospitalized were not statistically significant we speculate that this potential bias would be minor. Four, in the group of SARS-CoV-2 RT-PCR-negative children the possibility of a previous asymptomatic infection with subsequent virus clearance cannot completely be ruled out.

In conclusion, this study evidenced that saliva sACE2 concentration was significantly greater in SARS-CoV-2 RT-PCR-negative children than in positive children and convalescent adults. We also showed that the differences in salivary sACE2 concentration of the protein between groups were mostly attributable to short-length isoforms < 90 kDa. Release of adequate concentrations of sACE2 in saliva could have a protective effect from SARS-CoV-2. Further longitudinal studies should be conducted to confirm the constitutive nature of sACE2 release in saliva and its protective role against the virus.

## Supplementary Information

Supplementary Information 1.

Supplementary Information 2.

## Data Availability

The data that support the findings of this study are available from the corresponding author, PB, upon reasonable request.
