# Supplementary figures and images for: Association between soluble angiotensin-converting enzyme 2 in saliva and SARS-CoV-2 infection: a cross-sectional study
Source: Sci Rep. 2023 Apr 12;13:5985. doi: 10.1038/s41598-023-31911-2 (PMC10092936; doi:10.1038/s41598-023-31911-2)

**A**

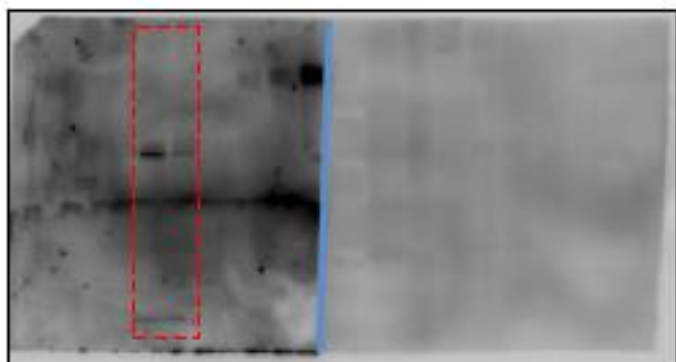

**B**

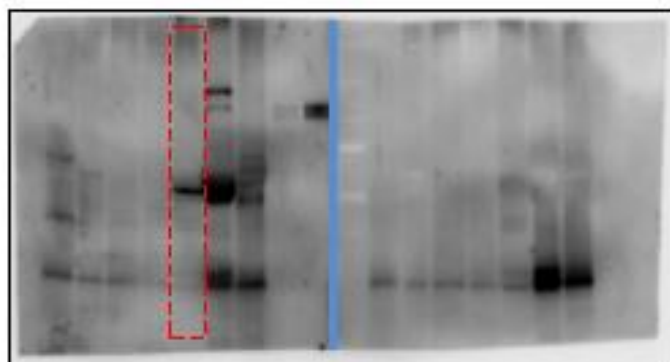

**C**

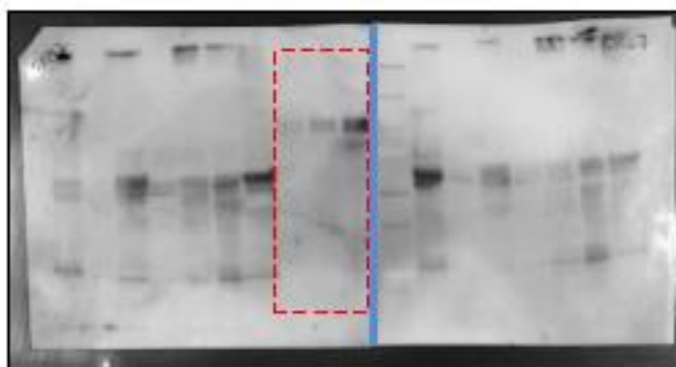

**D**

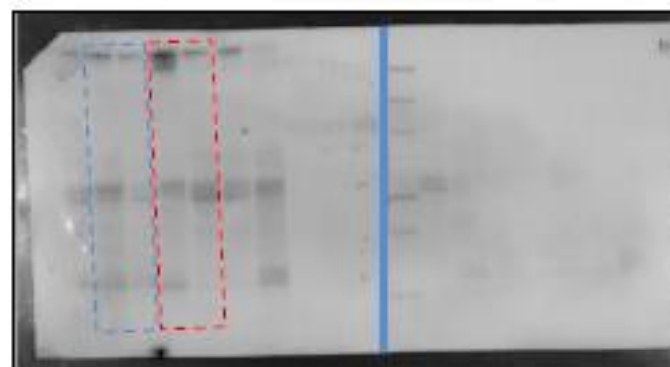

**E**

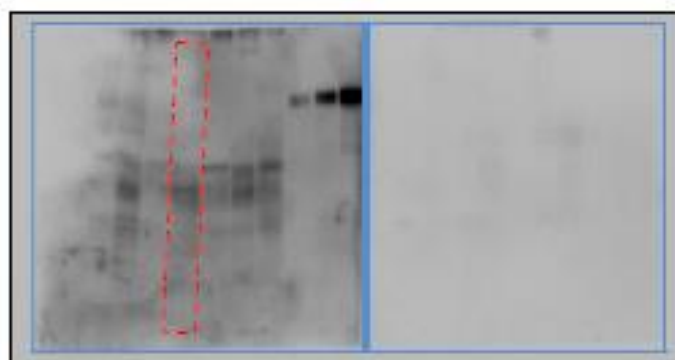

Supplement: Supplementary file 1 — Supplementary Information 1. [file 41598_2023_31911_MOESM1_ESM.pdf]
