# Supplementary material for: Association between soluble angiotensin-converting enzyme 2 in saliva and SARS-CoV-2 infection: a cross-sectional study
Source: Sci Rep. 2023 Apr 12;13:5985. doi: 10.1038/s41598-023-31911-2 (PMC10092936; doi:10.1038/s41598-023-31911-2)
Supplement: Supplementary file 2 — Supplementary Information 2. [file 41598_2023_31911_MOESM2_ESM.pdf]

Association between soluble angiotensin-converting enzyme 2 in saliva and SARS-CoV-2 infection: a cross-sectional study

Samuel Bru, PhD; Pedro Brotons, Iolanda Jordan, Laia Alsina, Desiree Henares, Reyes Carballar, Mariona Fernandez de Sevilla, Irene Barrabeig, Victoria Fumado, Bàrbara Baro, Joan Marc Martínez-Láinez, Juan J Garcia-Garcia, Quique Bassat, Albert Balaguer, Josep Clotet, Cristian Launes, Carmen Muñoz-Almagro

Supplementary Methods. Procedure for quantification of saliva sACE2 concentration and isoform identification

Saliva was incubated at 70^{0}C during ten minutes for virus inactivation and then centrifuged at 14,000 rpm and at 4ºC for one minute. A quantity of 30 µl of the supernatant were mixed with 6 µl of loading buffer (125mMTris-HCl at pH 6.8, 50% glycerol, 5% SDS, 0.25M DTT, and bromophenol blue) and boiled at 90°C for five minutes. Proteins were separated in Precast Protein Gels (4-15% Midi-PROTEAN® TGX^{TM}, BioRad) at 150-250V and transferred to PVDF membranes at 400 mA for one hour. Next, membranes were incubated with blocking solution (Tris Buffered Saline with Tween 20, pH=8 and 5% dry milk) at room temperature for 30 minutes and incubated with a diluted solution (1:1,000) of ACE2 antibody (66699-1-Ig, Proteintech) at 4°C overnight. This is a monoclonal mouse antibody that recognizes the ACE2 ectodomain. Next, membranes were washed with 50 ml of

TBS-T 3 times for ten minutes, incubated with a diluted solution (1:33,000-1:50,000) of Goat Anti-Mouse light chain Antibody, HRP conjugate (AP200P, Proteintech) at room temperature for one hour, and washed again with 50 ml of TBS-T at least 5 times for ten minutes. Immunoblots were developed using SuperSignal™ West Femto Maximum Sensitivity Substrate and images were acquired using ChemiDoc Imaging System (BioRad). Protein quantification was performed (Image Lab Software; BioRad) using different amounts of ACE2 as standard. The total protein concentration was calculated using the Bradford protein assay.
